# Synthesis of Bio-Based Polybenzoxazine and Its Antibiofilm and Anticorrosive Activities

**DOI:** 10.3390/ma16062249

**Published:** 2023-03-10

**Authors:** Chaitany Jayprakash Raorane, Thirukumaran Periyasamy, Rajesh Haldhar, Shakila Parveen Asrafali, Vinit Raj, Seong-Cheol Kim

**Affiliations:** School of Chemical Engineering, Yeungnam University, Gyeongsan 38541, Republic of Korea; chaitanyaraorane22@ynu.ac.kr (C.J.R.); thiru.kumaran999@gmail.com (T.P.); rajeshhaldhar.lpu@gmail.com (R.H.); shakilaasraf@gmail.com (S.P.A.)

**Keywords:** polybenzoxine, curcumin, biofilm, anticorrosion, *C. albicans*

## Abstract

*Candida albicans* are highly widespread pathogenic fungi in humans. Moreover, its developed biofilm causes serious clinical problems, leading to drug failure caused by its inherent drug tolerance. Hence, the inhibition of biofilm formation and virulence characteristics provide other means of addressing infections. Polymer composites (PCs) derived from natural products have attracted increasing interest in the scientific community, including antimicrobial applications. PCs are a good alternative approach to solving this challenge because of their excellent penetration power inside biofilms. The main objectives of this study were to synthesize a novel curcumin-based polybenzoxazine polymer composite (poly(Cu-A) PC) using Mannich condensation reaction and evaluate their potency as an antibiofilm and anticorrosive candidate against *C. albicans*. In addition, their anticorrosive efficacy was also explored. PC exhibited significant antibiofilm efficacy versus *C. albicans* DAY185 by the morphologic changing of yeast to hyphae, and>90% anticorrosive efficacy was observed at a higher dose of PC. These prepared PC were safe in vivo against *Caenorhabditis elegans* and *Raphanus raphanistrum*. The study shows that a polybenzoxazine polymer composite has the potential for controlling biofilm-associated fungal infections and virulence by *C. albicans*, and opens a new avenue for designing PCs as antifungal, anticorrosive agents for biofilm-associated fungal infections and industrial remediation.

## 1. Introduction

Polybenzoxazines are high-performance thermosetting resins produced by the polymerization of benzoxazine monomers produced by Mannich condensation from formaldehyde, phenol, and amine, which provides considerable flexibility as different amine and phenol precursors can be used. Furthermore, benzoxazine monomers undergo thermally induced ring-opening polymerization (ROP) to form polybenzoxazines. These polymers have many advantages, such as zero shrinkage upon curing, no release of volatile products, low water absorption, appreciable mechanical and thermal properties, and superior chemical and electrical resistance properties, and thus, they have many applications [1,2,3]. Curcumin is a polyphenolic compound available abundantly in turmeric (*Curcuma longa*). It is extensively used in the medicinal field as it functions as an antioxidant, antibacterial, antiviral, and anti-inflammatory agent [4,5]. Additionally, due to the existence of –OH groups in its structure, curcumin can be used in the synthesis of several monomers or can be made to blend with several polymers, where the –OH functional group is required [6,7].

The universal affliction of infectious diseases has substantially enhanced due to the evolving and re-emerging progress of resistant strains of microbes. Recent reports show that biofilm-producing bacterial strains are related to more than 65% of bacterial infections [8,9]. Such challenging infections necessitate strategic therapeutic and prophylactic drug intercessions. Within a matrix of extracellular polymeric substances (EPS), a consortium of microorganisms attaches to an abiotic or biotic surface called biofilm. The capability to develop biofilms is a significant virulence factor of numerous microbes. Therefore, elevated concentrations of antimicrobial agents, rapid medical intervention, and an alternative to infected devices are needed to manage biofilm infections [10,11].

The human body can be infected by various pathogenic agents such as viruses, fungi, and bacteria. Bacterial and fungal infections are the most common type of acute and chronic infections, causing worldwide morbidity [12]. *C. albicans* is an opportunistic pathogen and the primary cause of superficial, mucosal, and dermal fungal infections, especially in immunocompromised patients and individuals with inserted medical devices [13,14]. Mainly diseases produced by *C. albicans* are accompanied by the development of biofilms on the host or abiotic surface, causing high mortality and morbidity. Biofilm formation makes treatment challenging and highly resistant to current fungicidal drugs, sequentially leading to the use of higher doses of antifungal agents to treat an infection. In some cases, the use of higher amounts of antifungal agents can trigger serious difficulties, comprising liver and kidney injury [15].

In this study, a novel curcumin-based Pbz polymer composite (poly(Cu-A) PC) was synthesized to adopt the properties of curcumin into the Pbz network. The synthesized bio-based polybenzoxazine act as a nontoxic, eco-friendly green coating for antibiofilm formation and anticorrosion. The synthesized polymer composite (PC) was examined by differential scanning calorimetry (DSC) and Fourier-transform infrared spectroscopy (FTIR), and thermogravimetric analysis (TGA) was conducted to examine the thermal behavior of the poly(Cu-A) PCs. The produced polymer composite PCs were tested for their ability to constrain biofilm development through fungal strain *C. albicans* DAY 185. Antifungal and antibiofilm studies were performed to assess the impacts of the functionalized PCs on *C. albicans*. The *Candida* hyphae morphology to support the antibiofilm potency and microscopically hyphae of *C. albicans* and the fungal biofilm morphology were analyzed. The toxicity of poly(Cu-A) PCs was assessed with plant seed germination and nematode *Caenorhabditis elegans*. In addition, electrochemical studies were carried out to investigate the anticorrosive efficiency of poly(Cu-A) PCs for low-carbon steel (LCS). 

## 2. Materials and Methods

### 2.1. Experimental Section and Materials

Curcumin (95%), aniline, and paraformaldehyde (98.7) were acquired from Sigma–Aldrich (St. Louis, MO, USA). DMSO (98.8%), along with NaOH (98.9%), was provided by Samchun Pure Chemicals (Seoul, South Korea). For electrochemical studies, LCS was used. For each experiment, LCS was sanded with SiC sandpaper and granulated from 200 to 3000. For in vitro studies of poly(Cu-A) *C. albicans* strain, namely DAY185 (fluconazole-resistant) [16], was kindly offered by Prof. Jintae Lee, Yeungnam University, South Korea and was originally acquired from the KCCM (South Korea) (http://www.kccm.or.kr/) accessed on 15 December 2017. Potato dextrose agar (PDA) and broth (PDB) were used for sub-culturing *C. albicans*. Biofilm experiments were performed by overnight incubation at 37 °C by inoculating a single colony into 15 mL of PDB medium. At least two independent experiments were conducted.

### 2.2. Instrumentation Methods

Fourier transform infrared (FT-IR) spectra were found by using a Perkin Elmer MB3000 FTIR spectrometer (Waltham, MA, USA). The spectra were acquired at a resolution of 4 cm^−1^ in the IR range of 400–4000 cm^−1^. Samples were prepared by grinding with KBr and compressed to form discs. ^1^H NMR NMR (nuclear magnetic resonance) spectra were recorded using an Agilent NMR (VNS600, Santa Clara, CA, USA) at 600 MHz, and samples were dissolved in DMSO. To determine the T_m_ and T*_g_* and heat of curing of the monomers, DSC was used and was performed in a TA instrument (New Castle, DE, USA) Q_10_ model at a heating rate of 10 °C min^−1^ from ambient to 300 °C in N_2_ atmosphere using 8–10 mg of the sample. TGA thermograms were acquired using TA instruments SDT Q_600_ series thermogravimetric analyzer. All the runs were conducted in a nitrogen atmosphere with a gas flow rate of 30 mL/min. At a heating rate of 20 °C/min from ambient to 800 °C, all TGA experiments were conducted.

### 2.3. Synthesis of Curcumin-Based Bzo Monomer

**Scheme 1:**
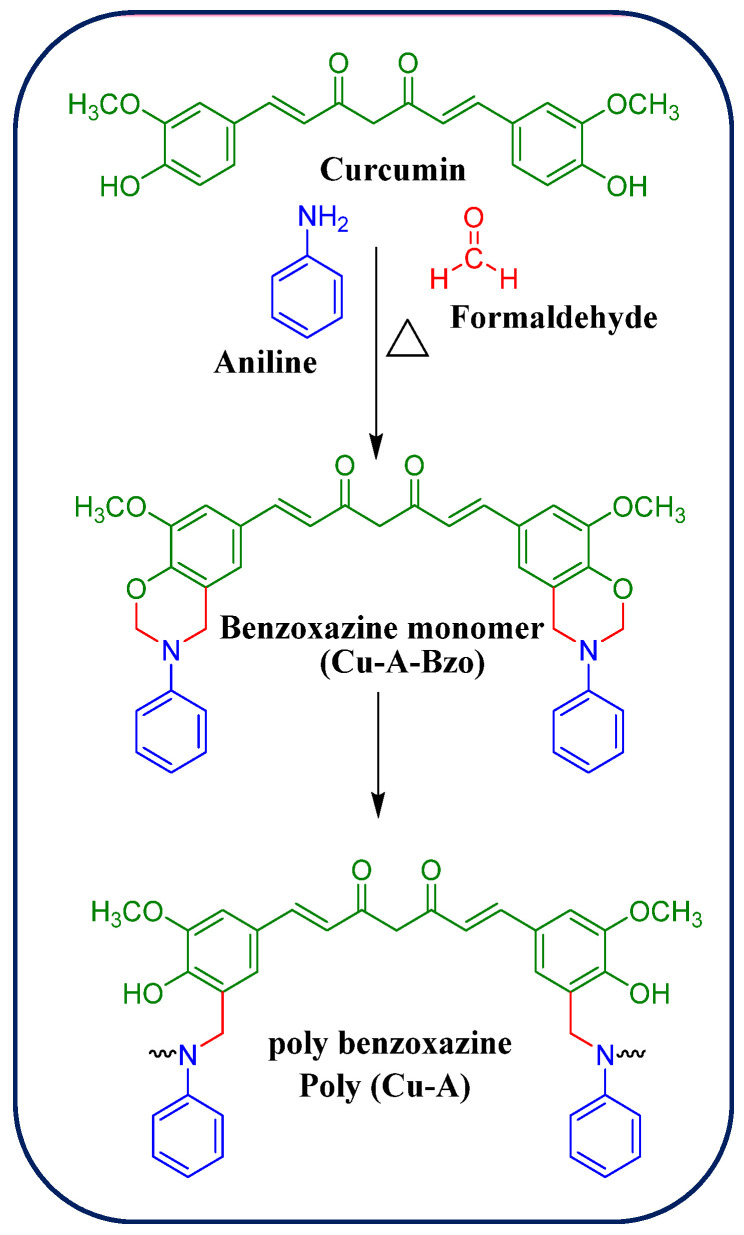
Synthesis of Cu-A-Bzo and poly(Cu-A).

Cu-A-Bzo was produced by Mannich condensation, as follows [16]. In a round-bottomed flask fitted with a reflux condenser, paraformaldehyde (1.2 g, 0.04 m) and DMSO (15–20 mL) were added and allowed to stir for a few minutes, maintaining the temperature at 70 °C. When paraformaldehyde started to dissolve in the solution, aniline (1.86 g, 0.02 m) and curcumin (3.68 g, 0.01 m) were taken separately to the stirring solution. The temperature of the solution was increased to 120 °C and kept under stirring for 5 h. After which, the reaction mixture was cooled to room temperature and poured into 1N NaOH solution to precipitate the benzoxazine monomer. Using DI water, the precipitate was washed 5 times and finally filtered and dried at 70 °C to produce a yellow-colored product with an 85% yield. The obtained product is denoted as Cu-A-Bzo (Scheme 1). Curcumin plays the role of phenolic moiety from a natural source and reacts with the amine group (of aniline) and formaldehyde to form the benzoxazine structure. As curcumin has two phenolic groups, a bifunctional benzoxazine monomer is formed, which further aids in increasing the crosslink density during Pbz formation.

### 2.4. Preparation of Polybenzoxazine [Poly(Cu-A)]

The synthesized benzoxazine monomer (Cu-A-Bzo) was polymerized to produce Cu-A-Pbz by placing a few grams of Cu-A-Bzo on a glass Petri dish and subjecting it to; 1 h at 100 °C, 1 h at 150 °C, 1 h at 200 °C, and 3 h at 250 °C (Scheme 1). The cured product, i.e., poly(Cu-A), was analyzed for various studies.

### 2.5. Electrochemical Studies

Electrochemical measurements were performed using a Corrtest CS2350 potentiometer (Wuhan, China) and CS Studio5 analysis software. A three-electrode set-up was used, viz. an LCS working electrode, a platinum counter electrode, and an Ag/AgCl reference electrode. The contact area between the LCS and electrolyte was 1 cm^2^ [17], and the LCS electrode was held at open circuit potential (OPC) for > 60 min after being submerged in 1 M HCl with or without Cu-A-Pbz [18]. The PDP was conducted at a scan rate of 1 mV/s in the vicinity of 1200 mV vs. OCP. The impedance measurements were conducted with an equal amplitude (10 mV) in the frequency range of 100 kHz to 0.1 Hz at room temperature [19,20,21]. Nyquist and Bode graphs were used to assess the findings’ corrosion performance. ZView Software was used to mimic the impedance behavior using an electrical equivalent circuit.

### 2.6. Antibiofilm Potency of Poly(Cu-A) against C. albicans

Biofilm assays were performed using the crystal violet staining method [22]. Briefly, overnight culture of *C. albicans* DAY185 was inoculated in PDB at a dilution ratio of 1:25, and PDB cultures in 96-well microtiter plates were treated with poly(Cu-A) at 0–100 μg/mL for 24 h at 37 °C. Biofilm development was verified by staining with 0.1% crystal violet for 30 min and frequently washed with distilled water; then, in each well, 300 μL of 95% ethanol was added. Using an Elisa microplate reader with (Biobase, Jinan city, China), the absorbance of each 96-well microtiter plate was noted at 570 nm. Biofilm assay was performed with two independent cultures in triplicates. According to Clinical Laboratory Standards Institute (CLSI) for yeast, [23] MIC was defined as the lowest concentration that inhibited cell growth. Briefly, freshly grown *C. albicans* cells were diluted for the optimum size of inoculum for MICs. Cation-adjusted Mueller–Hinton broth media were used in this study. Experiments were performed using at least two independent cultures.

### 2.7. Yeast Hyphae-Switch Assay

A yeast hyphae-switch assays were performed as previously described [24]. *C. albicans* DAY185 cells were suspended in PDB in 14 mL polypropylene tubes and treated or not treated with poly(Cu-A) (0–200 μg/mL) under static conditions for 24 h at 37 °C. Images were captured using an optical imaging system (Nikon Eclipse 50i, Seoul, South Korea).

### 2.8. Time–Kill Kinetics

Time-to-kill kinetic studies were performed, as previously described [25]. Briefly, overnight cultures of *C. albicans* in PDB (dilution 1:25) were incubated with 0–400 μg/mL of poly(Cu-A) for 2 h at 37 °C with shaking (240 rpm). At precise time intervals, aliquots of treated and untreated cells were collected and diluted in PBS and plated on PDA plates. After 24 h incubation of PDA plates at 37 °C, colony-forming units (CFUs) were counted and plotted against specific time intervals. The assay was conducted at least three times using two independent cultures.

### 2.9. Reactive Oxygen Species Assay

The ROS production in *C. albicans* was determined, as previously described [25,26]. *C. albicans* cells were grown overnight in PDB washed with PBS, resuspended in PBS at 10^5^ CFU/mL, and treated with poly(Cu-A) (0–100 μg/mL) or H_2_O_2_ (50 μg/mL; positive control) at 240 rpm for 1 h at 37 °C. Cells were then treated in the dark for 30 min at 37 °C with 2′,7′-dichlorofluorescein diacetate (5 μM; Sigma-Aldrich, USA). Growth was used to standardize fluorescence intensities (FI)/OD600. Optical densities and fluorescence of treated and untreated cultures were measured using a 3220 UV spectrometer (Optizen, Daejeon, South Korea) and multimode microplate reader JASCO-F-2700 (Hitachi, Tokyo, Japan), respectively. The excitation and emission slits were set to 5 nm, the excitation wavelength to 506 nm, and the emission intensities to 526 nm, respectively. Results are presented as the means of three independent experiments.

### 2.10. Architecture of C. albicans Biofilm

The phenotypic and biofilm architecture of *C. albicans* DAY185 on LCS were examined as previously described [22,24]. Briefly, LCS was sanded with SiC sandpaper (80–3000 grades), and sterile blocks (1.0 cm × 1.0 cm × 0.3 cm) were added to the wells of 6-well plates containing *C. albicans* in PDB and poly(Cu-A) (0–100 μg/mL) and incubated for 24 h, 37 °C. *C. albicans* cells adhered to LCS surfaces and were fixed by adding 100 μL of 1:1 mixture of formaldehyde (2%) and glutaraldehyde (2.5%) in each well. Cells were then fixed and stained with osmium tetroxide: PBS (1:1) and dehydrated using an ethanol series (50, 70, 80, 90, 95, and 100%). All samples were coated with platinum for 100 s, followed by SEM images observed by SEM (S-4800 SEM, Hitachi, Tokyo, Japan) at an accelerating voltage of 15 kV.

### 2.11. In Vivo Toxicity Assessment of Poly(Cu-A) on C. elegans

poly(Cu-A) toxicity was examined using synchronized adult *C. elegans* (*fer-15(b26);fem-1(hc17)*) nematodes, as previously described [27,28]. Briefly, 30–40 noninfected worms in every single well were added to a 96-well plate containing M9 buffer. The adult nematodes were treated with poly(Cu-A) at 0–500 μg/mL for seven days at 25 °C with gentle shaking. By using optical imaging equipment (Nikon Eclipse 50i, Daejeon, South Korea), the viabilities of worms were evaluated by exposing them to LED or UV LED lights [18] for 10–50 s. Three independent experiments were performed, and results are expressed as percentage nematode survivals.

### 2.12. In Vitro Seed Germination Toxicity Assay

The effects of poly(Cu-A) on *Raphanus raphanistrum* seed germination were analyzed using Murashige and Skoog agar plates, as previously described [29,30]. Overnight water-soaked seeds of *R. raphanistrum* seeds were used for seed germination toxicity assay. Seeds were sterilized by using 1 mL of 100% ethanol and 3% sodium hypochlorite solution treatment for 15 min. Then, sterilized seeds were put on agar plates containing 0.86 g/L Murashige and Skoog medium and poly(Cu-A) at 0–500 μg/mL with 0.7% bacto-agar. Plates were then incubated at room temperature for 7 days and photographed.

### 2.13. Statistical Analysis

All experiments were conducted at least in triplicate, and the results are expressed as the mean ± standard deviation. The Student’s *t*-test was used to determine the significance of differences between untreated and treated samples, and statistical significance was accepted for *p* values of <0.05 or <0.01, as indicated.

## 3. Results and Discussion

### 3.1. Structure Analysis of Cu-A-Bzo

**Figure 1:**
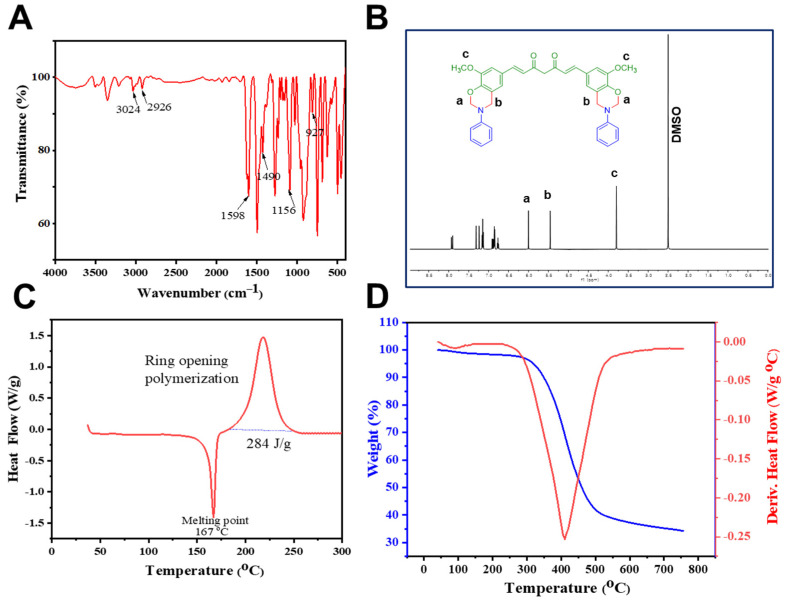
Characterization of Cu-A-Bzo and poly(Cu-A): (**A**) FTIR spectrum of Cu-A-Bzo; (**B**) ^1^H-NMR spectrum of Cu-A-Bzo; (**C**) DSC thermogram of Cu-A-Bzo; (**D**) TGA thermogram of poly(Cu-A).

The structure of the synthesized Cu-A-Bzo monomer was analyzed by FT-IR and NMR spectroscopy. The FT-IR spectrum of Cu-A-Bzo is shown in Figure 1A. The formation of the benzoxazine ring is identified by the absorption bands at 927 cm^−1^, corresponding to the oxazine ring vibrations of the –CH_2_ group. In addition to it, the asymmetric and symmetric stretching vibrations of C-O-C and C-N-C gave bands at 1274, 1092, and 1156 cm^−1^, respectively. Other vibrations of the curcumin moiety gave absorption bands at 1598 cm^−1^ due to the stretching vibration of the carbonyl group, and at 1490 cm^−1^, due to the aromatic C=C stretching vibrations. Moreover, the asymmetric and symmetric stretching vibrations of the aromatic –CH group were identified by weak bands at 3024 and 2926 cm^−1^, respectively. The structure of Cu-A-Bzo was further confirmed by ^1^H-NMR analysis, as shown in Figure 1B. The spectrum shows two singlets at 5.9 and 5.4 ppm, corresponding to the presence of oxazine ring protons, viz., O-CH_2_-N and Ar-CH_2_-N, respectively. The methoxy protons of the curcumin moiety produced a singlet at 3.6 ppm. Other aromatic protons resonated between 6.5 and 8.0 ppm. Thus, results confirmed the formation of curcumin containing benzoxazine monomer [31,32].

### 3.2. Curing Behavior of Cu-A-Bzo

The curing behavior of the Cu-A-Bzo monomer was analyzed by DSC. The DSC thermogram of Cu-A-Bzo is shown in Figure 1C and shows an endothermic and exothermic curve. The endotherm at 167 °C corresponds to the melting point of the Cu-A-Bzo, and the exothermic curve provides information on curing behavior. The onset of curing (T_onset_) starts at 184 °C with maximum curing (T_max_) at 218 °C and final curing (T_final_) at 249 °C. This means that a temperature of 250 °C is necessary for the complete polymerization of Cu-A-Bzo. Moreover, the amount of heat liberated during the curing/polymerization process is calculated to be 284 J/g. Therefore, the synthesized Cu-A-Bzo follows similar curing behavior w.r.t. bisphenol-A-based benzoxazine (BA).

### 3.3. Thermal Stability of Poly(Cu-A)

The thermal stability of poly(Cu-A) was analyzed by TGA (Figure 1D). The figure shows the weight loss of poly(Cu-A) with respect to temperature along with its derivative curve (DTG). The thermogram showed degradation occurred in one step and that poly(Cu-A) was thermally stable up to 300 °C. Its 10% degradation (T_10_) was found at 350 °C, and 50% degradation (T_50_) was found at 460 °C. The derivative curve shows maximum weight loss at 400 °C. The degradation of the polymer occurs between 300 and 500 °C, indicating a maximum steep in the curve. Moreover, a char yield of 35% was obtained after heating to 800 °C. These results showed poly(Cu-A) has a degradation profile similar to polybenzoxazine (Pbz) [33,34].

### 3.4. PDP and EIS Investigations

**Figure 2:**
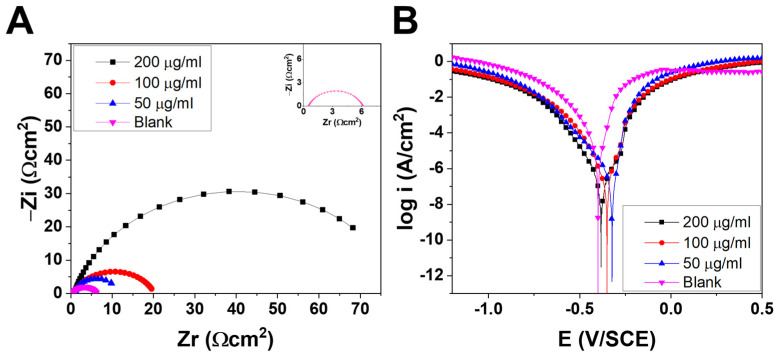
Electrochemical analysis of LCS: (**A**) Nyquist impendence curves; (**B**) potentiodynamic polarization curves in 1 M HCl at 293 K.

Potentiodynamic polarization experiments were conducted to understand the corrosion inhibition capabilities and the adsorption mechanism of the inhibitors on the LCS surface in a 1 M HCl medium. Figure 2A displays the polarization bending of LCS in this corrosive solution of 1 M HCl at 293 K in the presence and absence of poly(Cu-A) at different concentrations. PDP measurements provide critical factors, such as corrosion potential ($E_{corr}$), cathodic ($\beta_{c}$) and anodic ($\beta_{a}$) Tafel slope, corrosion current density ($i_{corr}$), etc. The following equation was used to compute the resistance to corrosion or corrosion inhibition efficiency [35].

(1) $$IE\left(\%\right)=\frac{i_{corr}-i_{corr}^{inh}}{i_{corr}}\times 100$$

where:

$i_{corr}$ = the corrosion current density without inhibitor;
$i_{corr}^{inh}$ = the corrosion current density with inhibitor.

The findings of LCS’s potentiodynamic polarization with and without the Cu-A-Pbz are shown in Table 1. Corrosion of the LCS was inhibited because poly(Cu-A) media binds to the surface [36]. As a result of this, anodic and cathodic Tafel slopes ($\beta_{a}$ and $\beta_{c}$, respectively) reduced the increasing poly(Cu-A) concentration. In addition, $i_{corr}$ (corrosion current density) resulted in increasing poly(Cu-A) concentration [37]. The anodic tafel slope ($\beta_{a}$) and cathodic Tafel slope ($\beta_{c}$) for blank shows 116.56 mV/dec and −171.86 mV/dec, which transfer to 112.21 mV/dec and −129.21 mV/dec at 200 µg/mL inhibitor concentration. During this investigation, most of the cathodic curves are in the form of Tafel lines, suggesting that the hydrogen reduction process takes place on the surface of the mild steel following an activation kinetic pattern. Moreover, for the four inhibitors, a pseudo-bridge is observed with a higher potential than 300 mV/Ag/AgCl (desorption potential or polarization potential). This phenomenon is caused by the desorption of these molecules on the steel surface by a strong polarization of the working electrode. The displacement of $E_{corr}$ is within the bracket of 85 mV with respect to the blank (399.57 mV), which suggests the mixed-type nature of the studied inhibitor. For the blank, the corrosion potential value is 399.57 mV, while at 200 µg/mL inhibitor concentration, it shows 382.79 mV. Additionally, it was noted that as poly(Cu-A) concentration increases, values of $i_{corr}$ (corrosion current density) continually drop [38]. This may be caused by the strong coordination connection that exists in the poly(Cu-A) compound between the free electrons of heteroatoms and the unoccupied d-orbitals of iron. For blank, the corrosion current density value is 11.25 mA/cm^2^, while at 200 µg/mL, it shows 0.83 mA/cm^2^. At a 200 µg/mL concentration, poly(Cu-A) had the most significantly lowered $i_{corr}$ value (830 µA cm^−2^). At this concentration, it displays 92.62% IE.

EIS spectra were drawn using Nyquist plots (Figure 2B) [35]. The results are shown in Table 1. The following equation was used to calculate corrosion inhibition effectiveness and other important factors [36]:

(2) $$IE\left(\%\right)=\frac{R_{ct}^{inh}-R_{ct}}{R_{ct}^{inh}}\times 100$$

where:

$R_{ct}^{inh}$ = the charge transfer resistance with inhibitor;
$R_{ct}$ = the charge transfer resistance without inhibitor.

LCS corrosion is represented by the capacitive loops, and it is controlled by the charge transfer mechanism of corrosion [39]. As shown in Figure 2B, the semicircles’ diameter grows as poly(Cu-A) is added to the corrosive medium. The greater the corrosion inhibition, the larger the loop’s diameter [40], and poly(Cu-A) had the greatest semicircle diameter at 200 µg/mL, indicating maximum corrosion resistance [41]. Table 1 shows that when the inhibitor concentration increased, $C_{dl}$ decreased and $R_{p}$ increased. The $R_{s}$ values show the solution resistance; for blank, it shows 0.54 Ωcm^2^, while at 200 µg/mL inhibitor concentration, it shows 0.85 Ωcm^2^. Polarization resistance also increases from 5.59 Ωcm^2^ (blank) to 79.07 Ωcm^2^ for 200 µg/mL inhibitor solution showing its extraordinary ability of corrosion resistance. $C_{dl}$ values dropped from 1335.80 µF/cm^2^ (blank) to 577.60 µF/cm^2^ for 200 µg/mL inhibitor concentration, showing its best adsorption abilities. These findings exhibit extraordinary surface adsorption of poly(Cu-A). The outcomes achieved by the EIS technique are in agreement with the PDP [42,43]. Poly(Cu-A) showed 92.93% hindrance at 200 µg/mL and $R_{p}$ (79.07 Ω cm^2^).

### 3.5. Antibiofilm Potency and SEM Analysis Poly(Cu-A) PCs Treated C. albicans

**Figure 3:**
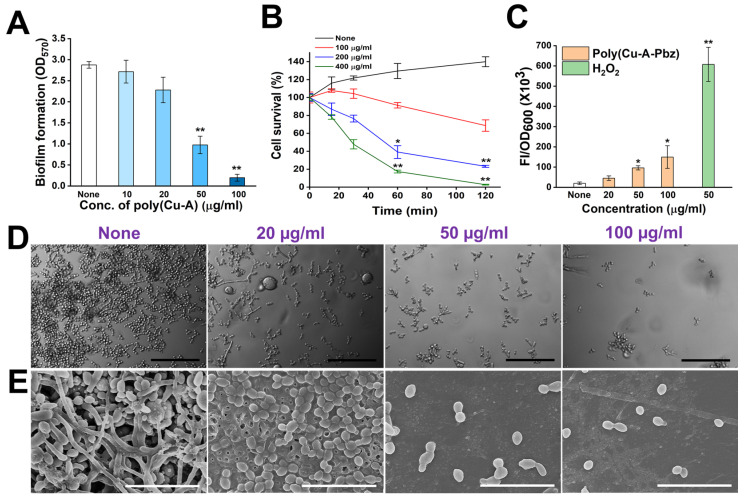
Evaluation of antifungal and antibiofilm potency of poly(Cu-A) compared to *C. albicans* DAY185: (**A**) biofilm formation of *C. albicans* DAY185 in the existence of poly(Cu-A); (**B**) rapid killing of *C. albicans* by poly(Cu-A), after incubation of 2 h with poly(Cu-A); (**C**) Poly(Cu-A)-induced ROS production by *C. albicans*; (**D**) hyphae were pictured after incubation over 24 h; (**E**) SEM analysis of *C. albicans* biofilm against poly(Cu-A). Scale bars: black = 100 µm, white = 20 µm. The findings are explained as means ± SDs of triplicate. The error bars indicate standard deviations. ** *p* < 0.01 and * *p* < 0.05.

A biofilm assay was used to investigate the antibiofilm potency of poly(Cu-A) against C. albicans. Treatments with poly(Cu-A) at 20, 50, or 100 µg/mL dose-dependently inhibited biofilm formation (Figure 3A). At 50 µg/mL poly(Cu-A) and incubation for 24 h poly(Cu-A) inhibited biofilm formation by >65%, and at 100 µg/mL, this inhibition increased to > 92%, and cell growth was only marginally affected. Furthermore, the MICs of poly(Cu-A) against *C. albicans* was 250 µg/mL. Optical microscopy was used to assess the effects of poly(Cu-A) PC on the *C. albicans* morphology. (Figure 3D). Non-treated *C. albicans* colonies containing large cell aggregations by pseudo hyphae were observed after incubation for 24 h. However, poly(Cu-A) PC substantially reduced cell aggregation (Figure 3D). SEM showed that poly(Cu-A) PC in PDB medium at 50 and 100 μg/mL suppressed the hyphae transition on the surface of LCS, as shown in Figure 3E. *C. albicans* contained a predominance of large hyphal cells in untreated biofilms, whereas poly(Cu-A) PC-treated biofilms were composed of yeast cells with rare hyphae. Additionally, the cell-aggregation and hyphal findings were harmonious in conjunction with the detected antibiofilm activity in the treatment group. Modified chitosan-based benzoxazine precursor and amino cellulose-based bio-films are capable of acting as antimicrobial and antifungal agents [44]. Renewable benzoxazine-based thermosets from cashew nuts [45] and biobased chitosan-grafted polybenzoxazine films are excellent antimicrobial agents [46]. As reported by Yadav et al. (2021), reversible labile linkages, expansion of chitosan galleries, and leaching of phenolic species from biobased polymer films led to improved antimicrobial activity [46]. Additionally, our research group synthesized bio-based Pbz films by blending chitosan with benzoxazine (Bzo) from curcumin and furfuryl amine, shown significant antibacterial and antibiofilm activity against Staphylococcus aureus and Escherichia coli [47]. These findings concurred with observed antibiofilm activities in the treatment group and showed that poly(Cu-A) prevented hyphal growth, biofilm formation, and the aggregation of *C. albicans* and had little impact on planktonic cell growth (Figure 3D,E).

### 3.6. Rapid Killing Activity

The time–kill kinetic study revealed poly(Cu-A) at 200 µg/mL needed 1 h to achieve a 60 ± 7.1% decrease in cell viability, whereas at 400 µg/mL, it killed more than 82 ± 1.6% of C. albicans cells within 30 min (Figure 3B) and at 400 µg/mL of poly(Cu-A) killed 97.2 ± 0.5% of cells in 2 h. These results suggest that the higher concentration of synthesized PC could be used to control the growth C. albicans DAY185. Several studies recently reported natural products containing polymeric and nonpolymeric composites used to eradicate human pathogens, such as C. albicans [44,48]. Additionally, benzoxazine-linked covalent organic framework materials have shown promising antimicrobial activity via postsynthetic cyclization [48,49].

### 3.7. ROS Assay

The generation of reactive oxygen species (ROS) causes oxidative stress in C. albicans and induces cytotoxicity and cell death [50]. Therefore, in the presence of poly(Cu-A), we investigated intracellular ROS production in C. albicans. Treatment with poly(Cu-A) considerably and dose-dependently enhanced ROS concentrations (Figure 3C). For example, at 50 or 100 µg/mL, poly(Cu-A) increased ROS levels by four- and seven-fold, respectively, whereas H_2_O_2_ (the positive control) at 50 µg/mL increased ROS intracellular production 29-fold. However, various research has been conducted on the generation of ROS by polyaniline nanocomposite, and polymeric composites worked as a versatile antifouling coating on implant surfaces against C. albicans [51,52].

### 3.8. In Vitro and In Vivo, and Environmental Toxicities of Poly(Cu-A)

**Figure 4:**
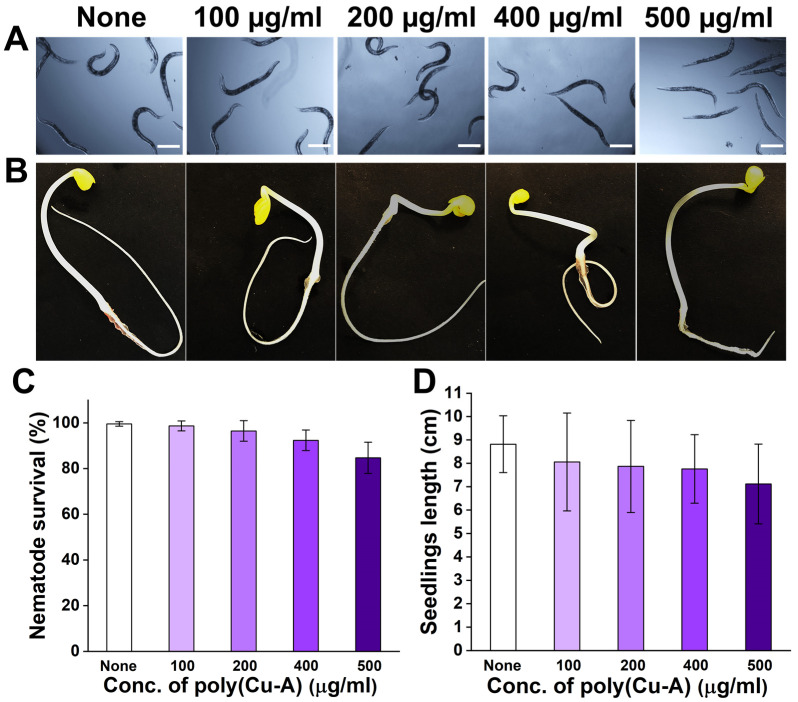
Poly(Cu-A) safety profile: (**A**) effects of poly(Cu-A) on C. elegans and (**B**) seed germination; (**C**) graph indicating the % of nematode survivals treated with poly(Cu-A) for five days; (**D**) the effects of poly(Cu-A) on seedlings length after seven days. Scale bar = 100 µm.

C. elegans was used to examine the toxic effects of poly(Cu-A). Microscopic examinations showed nematodes survived exposure to poly(Cu-A) at concentrations of 0–500 µg/mL for 7 days (Figure 4A,C). After 7 days trial, poly(Cu-A) treated nematode showed a similar trend to the non-treated controls (Figure 4A,C), confirming that poly(Cu-A) was nontoxic to worms and did not affect the survival rate. In particular, poly(Cu-A) at doses of <500 μg/mL did not influence survival or induce phenotypic changes in nematode morphology. For instance, >84% of worms were endured at different concentrations of poly(Cu-A), suggesting no toxicity effect by synthesized polymer composite. C. elegans is a premier toxicology model that has developed our understanding of cellular responses to synthetic, natural compounds or environmental pollutants and boasts robust genomic resources and high levels of genetic variation across the species [53]. There was a strong correlation between the toxicities between C. elegans and animals, which is the reason for assessing C. elegan’s toxicity [54,55].

The phytotoxicity and environmental impact of poly(Cu-A) were assessed using a seed germination experiment (Figure 4B). R. raphanistrum seeds were developed on Murashige and Skoog agar containing poly(Cu-A) in the range of 0–500 µg/mL. Poly(Cu-A) did not show any phenotypic changes to the germination of seeds for the three days, while at ≥200 µg/mL, the rate of R. raphanistrum germination was postponed after seven days (Figure 4B,D). Seed germination and seedling growth were slightly reduced by increasing the concentration of poly(Cu-A) from 100 to 500 µg/mL. These results indicate that poly(Cu-A) appears to be safe and environmentally friendly. As per recent reports polybenzoxazine based polymers, such as PAB/BX or bio-based phosphorus-containing benzoxazine [56,57], could be safer to discharge for agricultural fields and also to combat MDR microbes in the plant soil [29,30,31,32,33,34,35,36,37,38,39,40,41,42,43,44,45,46,47,48,49,50,51,52,53,54,55,56,57,58].

## 4. Conclusions

A curcumin functionalized benzoxazine monomer (Cu-A-Bzo) was successfully synthesized by Mannich condensation, as confirmed by FT-IR and NMR. The poly(Cu-A)polymer produced by thermally treating Cu-A-Bzo inhibited *C. albicans* biofilm formation and acted as an effective corrosion inhibitor. The poly(Cu-A) at 100 µg/mL concentration possesses >92% inhibition against *C. albicans*, which is higher than our previous work [57] based on arbutin-based polybenzoxazine with PEG-PPG-PEG (90.9% inhibition against *C. albicans* at 100 µg/mL concentration). Our results support the hypothesis that poly(Cu-A) might help prevent or treat *C. albicans* biofilm-associated infections. Interestingly, poly(Cu-A) also exhibited an exceptional ability to penetrate *C. albicans* biofilm in vitro. This is the first report on the synthesis of poly(Cu-A) PCs and their applications to the treatment of *Candida* biofilms and environmental remediation. Furthermore, the high MIC of poly(Cu-A) PC for *C. albicans* 250 µg/mL might allow it to be combined with fungicides to treat biofilm-associated chronic infections. The corrosion inhibition properties of poly(Cu-A) were effectively obtained in 1 M HCl corrosive media for LCS at 293 K. Depending on the acquired outcomes, it can be advised that after the measurement of the electrochemical investigation, the inhibition efficacy of poly(Cu-A) at 200 µg/mL was discovered to be approximately 93%. With every addition of inhibitory concentration, anodic and cathodic Tafel slopes essentially changed, which implies that the inhibitor molecule resists both the hydrogen evolution reaction and the metal dissolution process. Moreover, poly(Cu-A) at 200 µg/mL concentration has the largest semicircle, suggesting its best corrosion resistance performance. Thus, our results indicate poly(Cu-A) might be used to treat *C. albicans* biofilms or as an anticorrosive agent for further use.

## Figures and Tables

**Table 1:** The key parameters for LCS in 1 M HCl at 293 K obtained by electrochemical analysis.

Sample (µg/mL)	$R_{s}$ (Ω cm^2^)	$R_{p}$ (Ω cm^2^)	$C_{dl}$ (µF/cm^2^)	IE (%)	$-E_{corr}$ (mV)	$i_{corr}$ (mA/cm^2^)	$\beta_{a}$ (mV/dec)	$-\beta_{c}$ (mV/dec)	IE (%)
Blank	0.54	5.59	1352.80	-	399.57	11.25	116.56	171.86	-
50	0.73	11.32	951.64	50.62	322.51	5.58	85.06	114.24	50.40
100	0.74	19.37	667.80	71.14	350.35	3.01	94.72	145.76	73.24
200	0.85	79.07	577.60	92.93	382.79	0.83	112.11	129.21	92.62

## Data Availability

The raw/processed data required to reproduce these findings cannot be shared at this time as the data also forms part of an ongoing study.
